# A nano-silicate material with exceptional capacity for CO_2_ capture and storage at room temperature

**DOI:** 10.1038/s41598-018-30283-2

**Published:** 2018-08-07

**Authors:** Leide P. Cavalcanti, Georgios N. Kalantzopoulos, Juergen Eckert, Kenneth D. Knudsen, Jon Otto Fossum

**Affiliations:** 1 Institute for Energy Technology (IFE), Kjeller, Norway; 2 Centre for Materials Science and Nanotechnology (SMN), Department of Chemistry, University of Oslo, Oslo, Norway; 3 University of South Florida (USF), Tampa, USA; 4 Norwegian University of Science and Technology (NTNU), Trondheim, Norway

## Abstract

In order to mitigate climate change driven by the observed high levels of carbon dioxide (CO_2_) in the atmosphere, many micro and nano-porous materials are being investigated for CO_2_ selectivity, capture and storage (CCS) purposes, including zeolites, metal organic frameworks (MOFs), functionalized polymers, activated carbons and nano-silicate clay minerals. Key properties include availability, non-toxicity, low cost, stability, energy of adsorption/desorption, sorbent regeneration, sorption kinetics and CO_2_ storage capacity. Here, we address the crucial point of the volumetric capture and storage capacity for CO_2_ in a low cost material which is natural, non-toxic, and stable. We show that the nano-silicate Nickel Fluorohectorite is able to capture 0.79 metric tons of CO_2_ per m^3^ of host material - one of the highest capacities ever achieved - and we compare volumetric and gravimetric capacity of the best CO_2_ sorbent materials reported to date. Our results suggest that the high capture capacity of this fluorohectorite clay is strongly coupled to the type and valence of the interlayer cation (here Ni^2+^) and the high charge density, which is almost twice that of montmorillonite, resulting in the highest reported CO_2_ uptake among clay minerals.

## Introduction

A current major challenge in science and technology is the development of low cost materials with large CO_2_ capture and storage (CCS)^1^ capacity, retention ability and sorption selectivity^2^. Important factors for the choice of material in this context include its availability, environmental friendliness, non-toxicity, a low level of greenhouse gas emission during processing, material stability, production cost, CO_2_ storage capacity, energy of adsorption/desorption, sorbent regeneration, sorption kinetics and capacity per volume or per mass of host material. Many materials are being investigated including zeolites^3^, metal organic frameworks (MOFs)^4^, functionalized polymers^5,6^, activated carbons^7^ and nano-silicate clay minerals^8–10^. Also hybrid solutions using the best characteristics among all these absorbents are much investigated^11^. This work presents a quantitative study of the capture of CO_2_ by Fluorohectorite clay - a stable and low cost material - with different interlayer cations. Fluorohectorite clay is a modified hectorite clay, which is a species belonging to the smectite group^12^, classified as a 2:1 layered silicates, with layer periodicity of approximately 1 nm. The periodic structure includes an interlayer space where a cation is lodged, bonding the structure and balancing the overall charges. The group of smectites, commonly addressed in short as clays, also comprise more popular species like montmorillonite clays. The synthetic fluorohectorite, studied in the present work, has been demonstrated, in several publications by our group, to be a representative and clean model system of nano-silicate smectite clay minerals^13^. Synthetic nano-silicate clays contain significantly fewer impurities (e.g. carbonates, (hydr)oxides, silica, and organic matter) than natural clays and show a more homogenous charge distribution than their natural counterparts^14^, leading to well-defined intercalation states^15,16^. Intercalation of water in nano-silicate smectite clays occurs naturally and has been extensively studied with a wide range of techniques, among them neutron^17,18^ and X-ray scattering^13,19^. Recent experiments and simulations have shown that also CO_2_ can intercalate in smectite clays, both in supercritical^20^, and in gaseous/liquid form^21^. We have previously demonstrated^22^ that under certain conditions of pressure and temperature, fluorohectorite clays can capture a large amount of CO_2_ depending on the type of interlayer cation, and that Nickel-fluorohectorite clay (NiFh), in particular, will retain CO_2_ up to a temperature of 35 °C at ambient pressure^23^. The captured CO_2_ can subsequently be released by heating above this temperature. These conditions are highly relevant for mapping out and understanding the mechanisms involved in CO_2_ capture and retention by nano-silicates, either in geological formations, or in technological CO_2_ absorbent materials.

## Results and Discussion

**Figure 1:**
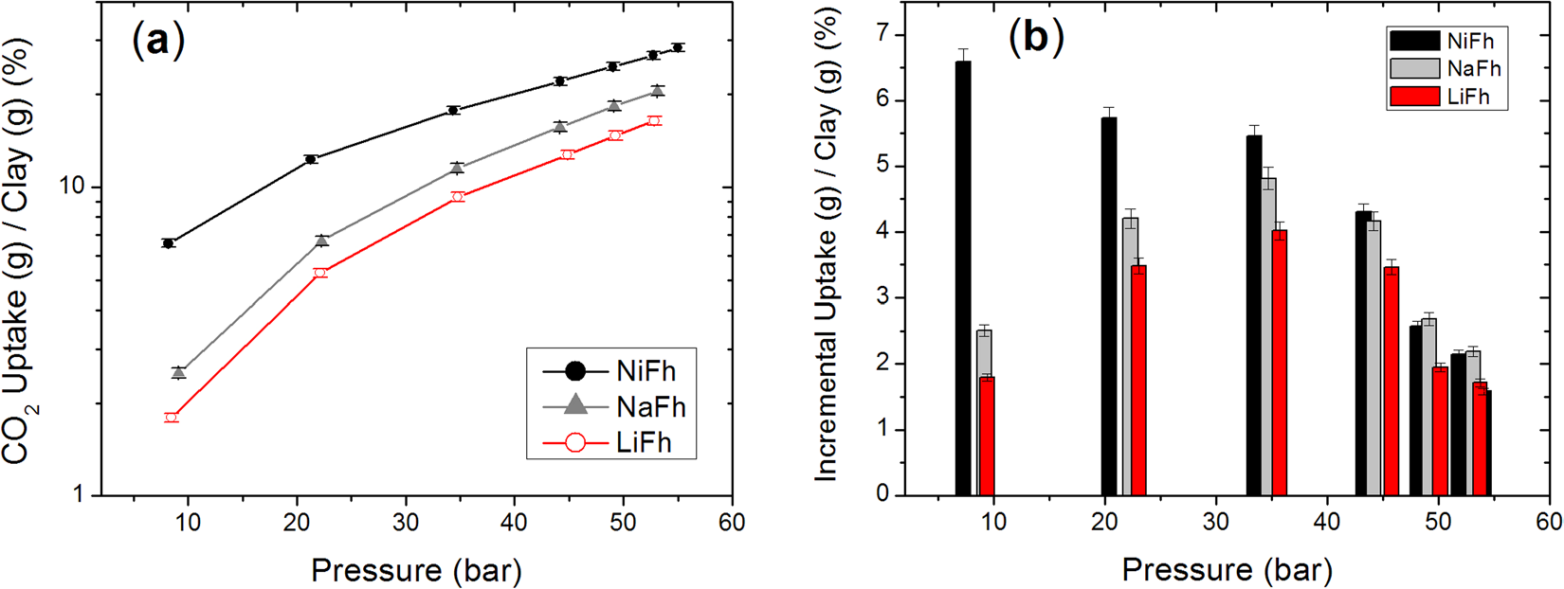
(**a**) Total uptake of CO_2_ into Fluorohectorite nano-silicate clay minerals determined for each pressure step at room temperature; (**b**) Incremental uptake of CO_2_ into Fluorohectorite clays for each pressure step.

We have utilized an accurately calibrated custom-made Sieverts apparatus for the quantification of CO_2_ storage capacity of the nano-silicate clay minerals. The setup is described in detail in the Methods section. The high-pressure CO_2_ uptake at room temperature (Fig. 1a) shows a maximum CO_2_ intercalation of 28% in weight for NiFh clay at the final pressure of 55 bar. This value is equivalent to 6.45 mmol of CO_2_ per gram of NiFh, which corresponds to 0.795 ton of CO_2_ per m^3^ of clay at 55 bar and room temperature, given the crystallographic density of the clay (2.8 g/ml). This result demonstrates that NiFh is able to intercalate one of the largest amounts of CO_2_ per volume of material of all porous materials reported in literature. Commercially available zeolite^3^ 13X can adsorb 0.35 ton/m^3^, the mesoporous carbon^7^ MPPY-4800 has been reported to uptake 0.93 ton/m^3^, whereas MOF-210^4^ exhibits a volumetric capacity of 0.72 ton/m^3^ of CO_2_ per volume of host material. Nevertheless, MOFs report the highest gravimetric capacity records because of their low density. Table 1 includes a comparison of exceptional results on CO_2_ uptake - showing both volumetric and gravimetric values - and specifically our results on high volumetric capacity clays, calling the attention to the diversity of characteristics among all materials that could be combined for a viable carbon capture and storage solution.

**Table 1:** CO_2_ intercalation values and clay parameters.

Material	Gravimetric capacity		Molar Uptake		Volumetric capacity	Final Pressure	Data
	Ratio (G) CO_2_ (g)/Material(g)	Fraction CO_2_ (g)/[CO_2_ (g) + Material(g)]	(G*m) CO_2_(mmol)/Material(g)	(d) Density (g/cm^3^)	(G*d) CO_2_ (ton)/Material(m^3^)	(bar)	
Clay NiFh (this work)	0.28	0.22	6.4	2.8	0.79 ± 0.02^†^	55	exp
Clay NaFh (this work)	0.21	0.17	4.7	2.8	0.58 ± 0.02^†^	53	exp
Clay LiFh (this work)	0.16	0.14	3.7	2.8	0.46 ± 0.02^†^	53	exp
Montmorillonite CTAB N2 (Stevens, 2013)	*0.11*	*0.10*	**2.42**	2.8	*0.31*	**1**	**exp**
Zeolite 13X (Cavenati, 2004)	*0.32*	*0.24*	**7.372**	**1.130**	*0.35*	**32**	exp
co-IonomIM-17% (Mg2^+^) (Rukmani, 2018)	*0.20*	**0.17**	**4.5**	**0.89**	*0.18*	**2**	**S**
MOF-aminoclay CuBTC@AC-2 (Chakraborty, 2016)	*0.24*	*0.19*	**5.4**	—	—	**1**	exp
mesoporous carbon MPPY4800 (Cox, 2017)	**2.42**	*0.71*	**54**	*0.38*	**0.93**	**50**	exp
MOF-210 (Furukawa, 2010; Sumida, 2012)	**2.87**	**0.74**	*65*	**0.25**	*0.72*	**50**	exp

**m** = 1000/Mw(CO_2_); ^†^Error propagation considering ± 2% uncertainty in mass of clay sample and ± 10% in volume; **exp** = experimental result; **S** = simulation; The values in **bold** are from the cited references; the values in *italic* were determined for the sake of comparison in this table.

The other clay types investigated here, NaFh and LiFh, are also found to be high capacity storage materials, with intercalation capacity of 21 wt % (corresponding to 0.58 ton/m^3^ or 4.7 mmol/g) and 16 wt % (corresponding to 0.46 ton/m^3^ or 3.7 mmol/g), both at a final pressure of 53 bar respectively. These numbers are higher than those found in a theoretical study on other clays^9^, for example, Na-Montmorilonite with a capacity of 1.5 mmol/g for a basal interlayer distance of 12 Å, which is comparable with the interlayer distance of our loaded samples as shown in Table 2. The volumetric capacity of LiFh was already studied by us and published elsewhere^23^. Here we report new results on LiFh for a comparison using the same experimental protocol (dehydration, incubation time and incremental steps in pressure) as used for NiFh. The results for NiFh are original and demonstrate a significant development forward regarding CO_2_ capture by a nano-silicate material.

**Table 2:** Fluorohectorite clay parameters.

Material	Mw	Cation	N**	N/cation ***	d_001_* not loaded	d_001_* loaded, 1st peak	d_001_* loaded, 2nd peak
Clay NiFh	791	Ni^2+^	5.1	8.5	10.9	12.1	13.1
Clay NaFh	773	Na^+^	3.6	3.0	9.6	12.3	—
Clay LiFh	754	Li^+^	2.8	2.3	10.3	11.9	—

*XRD data from previous work (Michels 2015) measured on samples of the same batch as in the present work; **Number of CO_2_ molecules captured per unit cell, ***N*** = (G/[Mw(CO_2_)/Mw(clay)]); ***Number of CO_2_ molecules captured per cation, ***N*/cation = **(***N***/x); x = 0.6 for Ni^2+^; x = 1.2 for Li^+^ and Na^+^.

NaFh and LiFh present very similar behavior in terms of the intercalation of CO_2_, as shown in Fig. 1. This suggests a similar interaction between the CO_2_ molecule and the Na^+^ and Li^+^ cations. They are both alkali metals of group 1 A with comparable ionic distribution in the nano-silicate clay interlayers. Previous results showed that after the intercalation into Fluorohectorites, CO_2_ is stable inside the nano-silicate clay material and can be released upon heating^23^, which indicates the formation of stable cation-CO_2_ complexes or cation solvation into a CO_2_ gas phase^24^. The size and structure of the complex might favor the stability and can be probed by X-ray diffraction (XRD). The XRD results from our previous work^23^, transcribed in Table 2, show that the interlayer separation occupied by CO_2_ for NaFh and LiFh increases with the ionic radius of the cation. Na^+^ has a larger ionic radius than Li^+^, and the loaded NaFh is found to have a larger interlayer separation than LiFh, which means additional room to accommodate a larger complex or higher coordination number. This could explain our finding that NaFh intercalates more CO_2_ than LiFh at the investigated pressures.

The total CO_2_ intercalated (Σ Ui) as a function of pressure is shown in Fig. 1a, while Fig. 1b shows the incremental amount (Ui) of CO_2_ intercalated at each step in pressure. The latter indicates that the process is driven towards a saturation limit, after which no additional intercalation will be possible. Since the incremental values have not reached zero at the maximum pressure used in this study, it is possible that CO_2_ intercalation is not yet at the full potential for this material. At this stage, despite presenting already a high uptake of CO_2_, further investigations into optimization of the CCS capacity of these materials may thus benefit from an extended pressure/temperature protocol as well as exploring the use of functionalized clays such as those prepared according to Breu’s group^25^. Reuse of the CO_2_-loaded clay materials is possible after releasing the CO_2_ by a combination of moderate heating and evacuation. This has been tested for NiFh, NaFh and LiFh (data not shown), with no sign of degradation of the material.

Table 1 shows the results for the CO_2_ gravimetric and volumetric capacity for the clays studied in the present work, compared with Montmorilonite^26^, Zeolite 13X^3^, functionalized polymers^5^, activated carbons^7^, MOF–aminoclay composites^11^ and MOF-210^4^ data from the literature. The final pressure varies according to the characteristics of the material. Some materials, like the fluorohectorite clays, have low uptake for flue gas but are not saturated at low pressure, allowing storage at high pressure. Table 2 shows the molar weight, number of CO_2_ molecules per unit cell (***N***) and the basal interlayer distance (d_001_) for each clay type.

The molecular formula per unit cell for NiFh, NaFh and LiFh is expressed by

$${{\rm{M}}}_{{\rm{x}}}({{\rm{Mg}}}_{6-{\rm{x}}}{{\rm{Li}}}_{{\rm{x}}}){{\rm{Si}}}_{8}{{\rm{O}}}_{20}{{\rm{F}}}_{4}$$

where x = 1.2 for LiFh (M = Li) and NaFh (M = Na) and x = 0.6 for NiFh (M = Ni)^27^. The number ***N*** is found to be 5 in the case of NiFh. This means more than 8 molecules of CO_2_ per Ni^2+^ cation, since NiFh has approximately 0.6 Ni^2+^ cations per unit cell^28^. The two other clays, LiFh and NaFh, contain 1.2 cation per unit cell, which gives a value for the ratio ***N/cation*** between 2 and 3.

**Figure 2:**
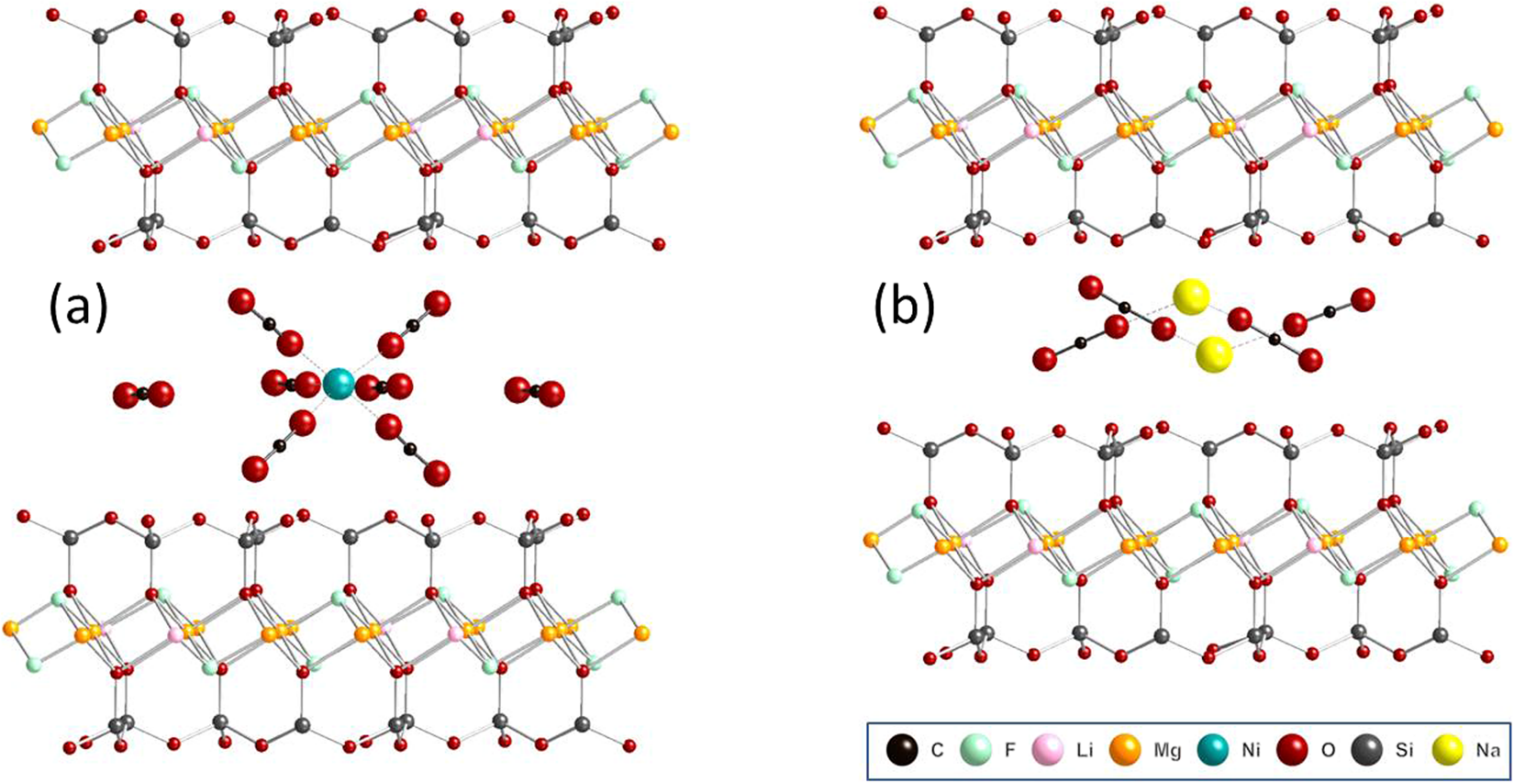
Suggested configuration for CO_2_-cation complexation inside Fluorohectorite clay interlayer: (**a**) 8 molecules of CO_2_ for each Ni^2+^ cation and (**b**) 2 molecules of CO_2_ for each Na^+^ cation. (The structures were built using the software CrystalMaker®).

The Fig. 2 shows possible structures of CO_2_-cation complexes assembled into NaFh and NiFh clay materials, respectively. These structures use as a starting point the crystal structure of water-containing clay, with cell parameters a = 5.2432 Å and b = 9.0870 Å, as published by Kalo *et al*.^8^, but here with the water molecules removed and substituted by another set of three atoms in the form of CO_2_. Our sketch of the CO_2_ intercalated fluorohectorite is intended to give a plausible picture of how the CO_2_ molecules could be arranged between the layers upon removal of water molecules. These hypothetical structures were derived by iterative process taking into account the nature of CO_2_-CO_2_, and CO_2_-host intercalations in a most qualitative way, while keeping interatomic distances from becoming too short. This was done without expanding the layer spacing from what it was for water. The three shortest CO_2_ oxygen-to-host-oxygen distances were 2.31, 2.53 and 2.607 Å, but these can, of course, be readily increased by increasing the layer spacing. We have started with an interlayer distance of 10 Å, yielding a layer periodicity of 15 Å (as in Kalo *et al*.^8^ for 2 water layers), which is close to the XRD results in the work of Michels *et al*.^23^ and the simulations in the work of Kadoura *et al*. (2012)^9^. The configuration of the intercalated complex is expected to be rather dynamic and the number of CO_2_ molecules around each cation is here estimated to be 2 for NaFh and 8 for NiFh based on the amount of material intercalated. For free Ni^2+^, the coordination number^29^ with CO_2_ can be as high as 6, and in Fig. 2(a) we assume this to be the case for intercalated Ni^2+^ as well. The binding of the two additional satellite molecules of CO_2_ could come from interaction with the clay layers.

In conclusion, we have in this work compared alternative materials with exceptional gravimetric or volumetric capacity for CO_2_ capture and storage. We have demonstrated that NiFh is able to capture 0.79 ton of CO_2_ per m^3^ of clay at 55 bar and ambient temperature. This is one of the highest volumetric capacities of all CO_2_ sorbent material reported to date. This result, combined with the diversity of characteristics of other high performance materials, could lead to new technological solutions for Carbon Capture and Storage (CCS). The storage capacity for fluorohectorite clays may well be further enhanced beyond the present results by utilizing functionalized clay minerals^8^. Moreover, it has also been suggested that clay materials can be used in gas separation, in particular, those of greenhouse gases such as methane^9^ from CO_2_.

Clay materials such as the synthetic Fluorohectorites investigated here are harmless^30^ and in addition, their production is expected to be substantially less expensive than other high performance porous synthetic materials considered for CO_2_ capture, such as MOFs whose upscaling is particularly challenging. Furthermore, clays are also proven to be highly stable when exposed to external mechanical stress or humidity on geological time scales.

Our results show that the type of clay-interlayer cation is critical for CO_2_ capture, whereas the clay nanolayers provide the large effective surface area responsible for the exceptional storage capacity. The remarkable capture capacity of NiFh is also related to (a) a high charge density, about twice that of Montmorillonite^28^, and (b) the presence of divalent Ni^2+^ that frees up additional space for CO_2_ intercalation, which results in a higher uptake per cation compared with the usual monovalent ions such as Na^+^. The internal surface area for fluorohectorite and montmorillonite was found to be 3 m^2^/g and 80 m^2^/g respectively in the work of Kaviratna *et al*.^28^ (using the fluorohectorite sample bought from the same batch as in the present work), which could be an indication that the CO_2_ uptake, in these two clays, is more dependent on the interlayer separation and interlayer cation than on the internal surface area. We believe that the main interaction mechanism responsible for the high CO_2_ affinity is direct dipolar electronic interaction with the interlayer cation. The physisorption is probably also, to some degree, influenced by the charge distribution of the clay layered structure.

Other aspects of sorption kinetics are under evaluation and DFT calculations of the intercalate CO_2_-cation structures are in progress using the initial model presented in Fig. 2 as starting point. This study will be combined with *in situ* spectroscopic characterization. Experiments using higher pressure, including liquid and supercritical CO_2_, are also planned. Our Sieverts apparatus allows for temperature measurements which will be important for future determination of heats of adsorption and comparisons with DFT calculations. The present work has been conducted on dehydrated clays; however it is of considerable interest to evaluate the sorption performance in the presence of water and selectivity for CO_2_ in the presence of CH_4_ and N_2_. The current results already make these materials highly interesting candidates for CO_2_ capture from dried industrial combustion gases.

## Methods

We have utilized a custom-made Sieverts apparatus for the quantification of CO_2_ storage capacity of the clay materials. The instrument has been designed and constructed for minimizing the experimental error and has been accurately calibrated according to procedures described in the literature^31^. CO_2_ gas was introduced to the reservoir and equilibrated for one hour, prior to allowing the expansion into the sample chamber and intercalation into the clay powder material. The number of CO_2_ gas molecules initially in the reservoir, n_i_ = P_i_V_i_/(Z_i_RT_i_), gas molecules was calculated using the compressibility of a real gas, Z(p,T), as a function of pressure and temperature with parameters from NIST tables as reported elsewhere^31^. For each pressure increment, the gas uptake was calculated from the difference n_f_ − n_i_, where n_f_ is the final number of gas molecules expanded to the total volume, V_t_ (sample chamber + reservoir): n_f_ = P_f_(V_t_ − V_s_)/(Z_f_RT_f_), where V_s_, sample volume, is the mass of sample per its bulk density. Prior to the gas sorption the clay powder was degassed for 20 hours at 120 °C under dynamic vacuum (down to 10^−7^ mbar), and the subsequent experiments were performed at 25 °C. The synthetic clays Nickel Fluorohectorite (NiFh), Sodium Fluorohectorite (NaFh) and Lithium Fluorohectorite (LiFh) and CO_2_ gas with purity >99.999% was used. LiFh was purchased from Corning Inc. NiFh and NaFh powder samples were obtained from the LiFh clay following a standard dialysis cation exchange protocol^32^ with no further analysis. Water intercalation in NaFh and NiFh samples, originated from the same LiFh batch that we have studied here, have been investigated by NMR spectroscopy (NaFh^29^), and TGA (NiFh^19^). The cation coordination numbers proposed from these results are in full accordance with the clay layer charge reported by Kaviratna *et al*.^28^.

The Sieverts apparatus had a reservoir volume of 16 ml and sample chamber of 13 ml. We have determined the number of CO_2_ molecules at the reservoir before and after expansion of the reservoir into the sample chamber. The whole process consisted of three stages: (A) degassing of sample and chambers, (B) accumulation of CO_2_ into the reservoir at a certain incremental step in pressure and, finally, (C) expansion of CO_2_ into the sample chamber. The total CO_2_ intercalation was determined as a sum of the intercalation amounts due to each incremental step in pressure. The CO_2_ intercalation was replicated for testing reproducibility. The amount of powder sample inside the chamber was around 2 g and the bulk density around 0.7 g/ml. The total intercalation period, steps 1 to 3, was uniform around 4 days for all samples. Previous experiments^22^ showed that the kinetics of intercalation is slower for NaFh compared to NiFh and LiFh, so we chose to expose all samples to the longest period. We expect that the intercalation time might reduce under different conditions, for example with supercritical CO_2_.
